# A Catalog of Rules, Variables, and Definitions Applied to Accelerometer Data in the National Health and Nutrition Examination Survey, 2003–2006

**DOI:** 10.5888/pcd9.110332

**Published:** 2012-06-14

**Authors:** Catrine Tudor-Locke, Sarah M. Camhi, Richard P. Troiano

**Affiliations:** Author Affiliations: Sarah M. Camhi, University of Massachusetts, Boston, Massachusetts; Richard P. Troiano, National Cancer Institute, Bethesda, Maryland.

## Abstract

**Introduction:**

The National Health and Nutrition Examination Survey (NHANES) included accelerometry in the 2003–2006 data collection cycles. Researchers have used these data since their release in 2007, but the data have not been consistently treated, examined, or reported. The objective of this study was to aggregate data from studies using NHANES accelerometry data and to catalogue study decision rules, derived variables, and cut point definitions to facilitate a more uniform approach to these data.

**Methods:**

We conducted a PubMed search of English-language articles published (or indicated as forthcoming) from January 2007 through December 2011. Our initial search yielded 74 articles, plus 1 article that was not indexed in PubMed. After excluding 21 articles, we extracted and tabulated details on 54 studies to permit comparison among studies.

**Results:**

The 54 articles represented various descriptive, methodological, and inferential analyses. Although some decision rules for treating data (eg, criteria for minimal wear-time) were consistently applied, cut point definitions used for accelerometer-derived variables (eg, time spent in various intensities of physical activity) were especially diverse.

**Conclusion:**

Unique research questions may require equally unique analytical approaches; some inconsistency in approaches must be tolerated if scientific discovery is to be encouraged. This catalog provides a starting point for researchers to consider relevant and/or comparable accelerometer decision rules, derived variables, and cut point definitions for their own research questions.

## Introduction

The National Health and Nutrition Examination Survey (NHANES) is a publicly available data resource that provides information from self- or proxy reports of health conditions and behaviors and biomedical data for a sample representing the US civilian noninstitutionalized population (www.cdc.gov/nchs/nhanes.htm). NHANES is administered in 2-year data collection cycles; the Physical Activity Monitor (PAM) component was introduced in the 2003–2004 and 2005–2006 cycles to collect accelerometer-based measures of physical activity among participants aged 6 years or older. During these 2 cycles, an ActiGraph model 7164 accelerometer (ActiGraph, LLC, Pensacola, Florida) was provided to ambulatory participants, representing the first time that a surveillance study collected accelerometer measures on a US representative sample.

The uniaxial accelerometer measured and recorded vertical acceleration as “activity counts.” The device also recorded “steps” by using a proprietary signal-filtering algorithm. These 2 related quantities measure physical activity movement associated primarily with locomotion. A 1-minute time interval, or “epoch,” was used in NHANES. Data for activity counts and steps were recorded during each epoch for up to 1 week. Both activity count and step data were released for the 2005–2006 NHANES cycle, but because of missing step data on a portion of the sample in the 2003–2004 cycle, only activity count data were released for that cycle. The data are available from http://www.cdc.gov/nchs/nhanes.htm; the National Cancer Institute (NCI) offers SAS (SAS Institute, Inc, Cary, North Carolina) syntax for analyzing the data at http://riskfactor.cancer.gov/tools/nhanes_pam/. The syntax facilitates the editing of invalid and unreliable intensity values (defined by NCI) and summarizes derived variables that describe the duration of nonwear periods and activity bouts of moderate, vigorous, and moderate-to-vigorous intensities. The NCI website acknowledges that “users can modify these programs to examine other issues, such as alternate definitions of valid data, monitor wear periods, or activity bouts.”

The release of PAM data in 2007 provided researchers a unique opportunity to study objectively measured physical activity on a large and representative US sample and relate it to a range of other health-related variables. Numerous studies using the data have been published, but these studies have treated, analyzed, and reported the data by using myriad accelerometer decision rules, derived variables, and cut point definitions. A catalog of these rules, variables, and definitions is needed so that researchers can begin to work toward more standardized and comparable data. The objective of this study was to catalogue the accelerometer decision rules, derived variables, and cut point definitions used in studies on PAM data published since 2007.

## Methods

### Data sources

We conducted an advanced English-only literature search of original research articles in PubMed by using the key terms “activity monitor” or “ActiGraph” or the wildcard term “acceleromet*” in addition to “NHANES” or “National Health and Nutrition Examination Survey.” We searched articles published from January 1, 2007, through December 31, 2011. We used the following search strategy: (“activity monitor” OR ActiGraph OR acceleromet*) AND (NHANES OR “National Health and Nutrition Examination Survey”) AND English[Language] AND (“2007/01/01”[Date of publication]: “2011/12/31”[Date of publication]). We included forthcoming and “epub ahead of print” articles and updated the search on February 10, 2012. We found 74 articles that met our search criteria. One author (R.P.T.) identified 1 other published study, prepared for a special conference, not indexed in PubMed (1) bringing the initial search total to 75 articles.

### Study selection

Twenty-one articles (28%) did not directly analyze PAM data, and they were eliminated; the remaining 54 articles (72%) were included in this review.

### Data extraction

The first author read and abstracted the following details from each identified article: 1) citation; 2) purpose of study; 3) PAM data collection cycle(s) analyzed (ie, 2003–2004 and/or 2005–2006); 4) study sample size and age of participants in sample; 5) whether investigators reported using the NCI-supplied SAS syntax; 6) rules for defining nonwear time (ie, time that the accelerometer was not likely worn), a valid day (ie, the minimum number of wear-hours required to be considered representative of a day’s behavior), and the minimum number of valid days required for a participant to be included in the analysis; 7) accelerometer-derived variables (eg, activity counts/day, time spent in moderate-intensity activity, steps in vigorous-intensity activity); and 8) cut point definitions used for each accelerometer-derived variable (ie, values used to categorize continuous data). The second author verified the details independently. Discrepancies were discussed and consensus achieved. The results were tabulated to facilitate comparison among studies. We made no attempt to contact the articles’ authors to obtain unreported information or clarify writing; data extraction was made on face value.

## Results

The purpose of the 54 articles varied (Table 1); they represented, for example, descriptive analyses (2-5), methodological analyses (1,6,7), and inferential analyses (8-10). Eighteen studies used the NHANES 2003–2004 cycle, 15 used the 2005–2006 cycle, and 21 combined data from both cycles. Sample sizes ranged from 103, representing prostate cancer survivors (11), to 6,329, representing participants with 1 or more days of wear in the 2003–2004 cycle (2,3). Fourteen studies focused primarily on children and/or adolescents (through age 19 y), 33 on adults (including 1 study on all participants aged ≥16 y), 2 on older adults, and 3 on all ages (ie, ≥6 y). Two reported only the mean age of cancer survivors.

**Table 1:** Studies Published Since 2007 (or Forthcoming) on Physical Activity Monitor Data, National Health and Nutrition Examination Surveys, 2003–2004 and 2005–2006

Reference	Purpose	Years of Study	Study Sample Size	Age of Study Participants, y
Troiano and Dodd (1)	Compare self-reported PA to objectively measured PA	2003–2004	3,087	≥20

Troiano et al (2)	Describe objectively measured PA	2003–2004	4,867 with ≥4 d, 6,329 with ≥1 d	≥6

Matthews et al (3)	Describe objectively measured time in sedentary behaviors	2003–2004	6,329	6–85

Tudor-Locke et al (4)	Describe objectively measured step-defined PA in children and youth	2005–2006	2,610	6–19

Tudor-Locke et al (5)	Describe peak stepping cadence in adults	2005–2006	3,522	≥20

Fan et al (6)	Validate self-reported PA for cholesterol control	2003–2004	789	≥18

Tudor-Locke et al (7)	Examine effects of wear time on accelerometer-derived variables	2005–2006	3,744	≥20

Mark and Janssen (8)	Examine dose-response relationship between objectively measured PA and blood pressure in children and adolescents	2003–2004	1,170	8–17

Sisson et al (9)	Examine associations between steps/d and metabolic syndrome and cardiovascular risk factors	2005–2006	1,446	≥20

Lynch et al (10)	Examine relationship of objectively measured PA and sedentary time with adiposity in breast cancer survivors	2003–2004 and 2005–2006	111	Mean 69.2

Lynch et al (11)	Examine the relationship between objectively measured PA, sedentary time, and waist circumference of prostate cancer survivors	2003–2004 and 2005–2006	103	Mean 75.4

Tudor-Locke et al (12)	Describe objectively measured step-defined PA	2005–2006	3,744	20–85

Tudor-Locke et al (13)	Compare objectively measured PA and inactivity profiles in normal-weight, overweight, and obese US men and women	2005–2006	3,522	≥20

Clark et al (14)	Examine the relationship between self-reported television viewing time and accelerometer-determined total sedentary time	2003–2004, and 2005–2006	5,738	≥20

Healy et al (15)	Examine the relationship between accelerometer-determined sedentary time and cardiometabolic health	2003–2004 and 2005–2006	4,757	≥20

Tucker et al (16)	Assess self-reported and objectively measured PA relative to 2008 Physical Activity Guidelines for Americans	2005–2006	3,082	≥20

Tudor-Locke et al (17)	Examine relationship between steps/d and other accelerometer-derived variables	2005–2006	3,744	≥20

Camhi et al (18)	Describe accelerometer-determined lifestyle activities and relationship with MVPA	2005–2006	3,744	≥20

Lynch et al (19)	Examine relationship between objectively measured PA, sedentary time, and biomarkers of breast cancer risk	2003–2004 and 2005–2006	1,024	≥20

Tudor-Locke et al (20)	Describe patterns of stepping cadence in adults	2005–2006	3,744	≥20

Evenson et al (21)	Describe objectively measured PA and sedentary behavior in adults 60 years or older	2003–2004 and 2005–2006	2,630	≥60

Winkler et al (22)	Compare methods of identifying sedentary time using automated estimates of accelerometer wear time	2003–2004	4,741	≥20

Vallance et al (23)	Examine the relationship between objectively measured PA, sedentary time, and depression	2005–2006	2,862	≥20

Peart et al (24)	Assess association between objectively measured PA, diet, sedentary behaviors, and overweight and obesity in US youth	2003–2004 and 2005–2006	2,638	12–19

Lee et al (25)	Examine the relationship between socioeconomic status and acculturation on objectively measured MVPA among Mexican American adolescents	2003–2004	322	13–19

Gortmaker et al (26)	Examine changes in child/adolescent PA by race/ethnicity between NHANES cycles	2003–2004 and 2005–2006	3,381	6–19

Smith et al (27)	Compare objectively measured PA levels among 5-year cancer survivors with those with no history of cancer	2003–2004 and 2005–2006	Not reported	≥20

Yang et al (28)	Examine relationship between receiving health care provider’s recommendation and adherence to healthy lifestyle among adults with prediabetes	2005–2006	2,853	≥20

Bankoski et al (29)	Examine the relationship between accelerometer-determined sedentary time and metabolic syndrome independent of PA	2003–2004 and 2005–2006	1,367	≥60

Belcher et al (30)	Describe objectively measured PA by race/ethnicity, age, sex, and weight status in youth	2003–2004 and 2005–2006	3,106	6–19

Mendoza et al (31)	Examine the relationship between active commuting to school and objectively measured PA and adiposity	2003–2004	789	12–19

Luke et al (32)	Examine the relationship of objectively measured PA with cardiovascular risk factors	2003–2004, and 2005–2006	3,370	20–65

Van Domelen et al (33)	Examine the relationship between employment and objectively measured PA	2003–2004	1,826	20–60

Holman et al (34)	Determine whether sporadic vs bout accumulation of MVPA was more strongly associated with cardiometabolic risk in children and youth	2003–2004 and 2005–2006	2,754	6–19

Loprinzi et al (35)	Examine association between objectively measured PA and C-reactive protein	2003–2004	4,555	≥6

Strath et al (36)	Describe objectively measured MVPA accumulation in bouts/nonbouts in relation to obesity	2003–2004	3,272 Waist circumference analysis; 3,250 body mass index analysis	≥18

Janney et al (37)	Examine relationship between objectively measured PA levels and use of mental health services	2003–2004	3,809	18–85

Metzger et al (38)	Describe patterns of objectively measured PA	2003–2004	3,802 ≥1 d, 3,462 ≥3 d	20–85

Mark and Janssen (39)	Compare MVPA bouts vs nonbouts in predicting overweight in youth	2003–2004 and 2005–2006	2,498	8–17

Hawkins et al (40)	Examine objectively measured PA among sex, age, and racial/ethnic groups	2003–2004	2,688	≥18

Metzger et al (41)	Examine patterns of objectively measured PA associated with metabolic syndrome	2003–2004	1,620	20–85

LeBlanc and Janssen (42)	Determine dose-response relationship between objectively measured PA and dyslipdemia in youth	2003–2004, and 2005–2006	1,235	12–19

Hagströmer et al (43)	Compare objectively measured PA between Sweden and United States	2003–2004	2,925	18–75

Ham and Ainsworth (44)	Describe disparities in objectively measured PA	2003–2004	3,043	≥18

LeBlanc and Janssen (45)	Examine differences between objective and self-reported MVPA in youth	2003–2004 and 2005–2006	2,761	12–19

Atienza et al (46)	Examine the independent associations of self-reported and objectively measured MVPA with physiologic and anthropometric biomarkers	2003–2004 and 2005–2006	5,797	≥20

Carson and Janssen (47)	Examine the relationship between sedentary behavior and cardiometabolic health in children and adolescents	2003–2004 and 2005–2006	2,527	6–19

Mark et al (48)	Explore effects of objectively measured PA intensity and incidental movement on body fat in children and youth	2003–2004	1,165	8–17

Hawkins et al (49)	Examine the relationship between objectively measured PA intensity and kidney function	2003–2004 and 2005–2006	2,117	≥18

Camhi et al (50)	Examine the relationship between accelerometer-determined lifestyle activities and cardiometabolic health	2005–2006	1,371	≥18

Tudor-Locke et al (51)	Compute a steps/d translation of time in MVPA	2005–2006	1,197	≥20

Evenson et al (52)	Describe objectively measured PA and sedentary behavior in pregnant women	2003–2004 and 2005–2006	359	≥16

Chasens and Yang (53)	Examine the relationship between insomnia and objectively measured PA in adults with prediabetes	2005–2006	958	≥20

Mendoza et al (54)	Examine the relationship of objectively measured MVPA and pediatric metabolic risk	2003–2004, 2005–2006	2,155	6–19

Abbreviations: PA, physical activity; MVPA, moderate- to vigorous-intensity PA; NHANES, National Health and Nutrition Examination Survey.

Twenty-four studies reported using the NCI-supplied SAS syntax (1,2,4,5,7,9,10,12-28). Eight studies (11,29-35) cited previous work, notably the first published study (2), that used the SAS syntax. The remaining 22 studies did not attribute their decision rules to another source (3,6,8,36-54). Most studies (42 of 54) defined nonwear time as 60 minutes or more of consecutive zeros, with or without allowance for interruptions, variously defined (Table 2). One study defined 10 minutes or more of identical consecutive nonzero counts as missing data (38). Seven studies that focused on children or adolescents or both defined nonwear time as 20 minutes or more of consecutive zeros (8,39,42,45,47,48). Four studies did not define a valid day; however, 2 of these studies may have implemented decision rules embedded in the NCI-supplied SAS syntax, which they reported using. Regardless, 49 of 54 studies defined a valid day as 10 hours or more of wear. The minimum number of valid days required for a participant to be included in the analysis varied; 17 studies required a minimum of 1 day; 23 studies required a minimum of 4 days; and 10 required a minimum of 4 days, including 1 weekend day.

**Table 2:** Rules for Data in Studies Published Since 2007 (or Forthcoming) on Accelerometer Data, National Health and Nutrition Examination Surveys, 2003–2004 and 2005–2006

Rule	No. of Studies	References
**Nonwear (time that the accelerometer was not likely worn) or missing**		
≥60 min of consecutive zeros	11	(5,20,22,24,27,36-38,40,41,49)
≥60 min of consecutive zeros with allowance for up to 2 min up to 100 activity counts/min	25	(1-4,6,7,9,12,13,15-18,21,22,26,29-33,43,44,46,52)
≥60 consecutive zeros with allowance for up to 2 min <50 activity counts/min	5	(10,11,14,19,22)
≥60 consecutive zeros with allowance for interruptions	1	(23)
≥10 min identical consecutive nonzero counts	1	(38)
≥20 min of consecutive zeros	7	(8,34,39,42,45,47,48)
Not reported^a^	7	(25,28,35,50,51,53,54)
**Valid day (minimum no. of wear-hours required to be considered representative of a day’s behavior)**		
10 h	49	(1-20,22-26,28-37,39,40,42-51,53,54)
Length of time that 70% of sample wore the accelerometer, multiplied by 70%	1	(52)
Not reported^a^	4	(21,27,38,41)
**Minimum no. of valid days required for a participant to be included in the analysis**		
All (missing data imputed)	1	(41)
1	17	(2-7,9,12,13,17,18,20,22,38,50,51,54)
2	1	(53)
3	1	(21,38)
4	23	(1,2,14,16,22-26,29-33,35-37,40,43,46,49,52,54)
4, including 1 weekend day	10	(8,15,19,34,39,42,44,45,47,48)
Not reported^a^	5	(10,11,27,28)

^a^ Decision rules embedded in the National Cancer Institute–supplied SAS syntax may have been used.

Studies on adults typically presented multiple accelerometer-derived variables (Table 3). Definitions differed for some similarly named variables. For example, some studies defined time in sedentary behavior as less than 100 activity counts per minute; others defined it as less than 260 activity counts per minute. Time in light intensity was defined as 100 to 759 activity counts per minute, 100 to 573 activity counts per minute, 100 to 1,951 activity counts per minute, 100 to 2,019 activity counts per minute, 260 to 1,951 activity counts per minute, and 500 to 2,019 activity counts per minute. Time in moderate- to vigorous-intensity physical activity (MVPA) was defined as 500 or more activity counts per minute, 574 or more activity counts per minute, 760 or more activity counts per minute, 1,000 or more activity counts per minute, 1,500 or more activity counts per minute, 1,952 or more activity counts per minute, 2,000 or more activity counts per minute, or 2,020 or more activity counts per minute. Time in MVPA was sometimes considered as any minute above the cut point and at other times only as minutes within a bout of 10 minutes or more (which may or may not have allowed for an interruption of 1 or 2 minutes below the cut point). Step data were reported in 2 ways: 1) in a raw or uncensored format (ie, not adjusted in any way) and 2) following a process of censoring steps from any minute with less than 500 activity counts per minute. (The latter process was designed to interpret the higher values of accelerometer-based step data against lower pedometer-based scales.) Physical activity levels were categorized according to a step-defined graduated index. Additional accelerometer-derived variables included time in incremental cadence (steps per minute) bands and peak cadence indicators (defined as the highest level of physical activity, or natural best effort, measured during a given day).

**Table 3:** Accelerometer-Derived Variables and Cut Point Definitions Used in Studies Published Since 2007 (or Forthcoming) on Accelerometer Data for Adults, National Health and Nutrition Examination Surveys, 2003–2004 and 2005–2006^a^

Accelerometer-derived variable	Cut point definition	Reference
Activity counts/d	Sum of daily activity counts	(13)
Mean activity counts/min	Sum of daily activity counts/number of min worn	(2,6,13,21,30,32,33,35,43,52)
Mean counts/min of most intense 10 min/wk	≥2,020 Activity counts/min	(6)
Time in sedentary behaviors (any min)	<100 Activity counts/min	(3,7,10,11,13-15,17,19,21-23,29,30,33,43,49,52)
Steps in sedentary behaviors (any min)	Steps detected <100 activity counts/min	(12)
Time in sedentary behaviors (>5-min bouts)	<100 Activity counts/min	(29)
Time in sedentary behaviors (any min)	<260 Activity counts/min	(37,40)
Proportion of sedentary time	Proportion of valid wear time at <100 activity counts/min	(29,33)
Stillness	Average intensity during time <100 activity counts/min	(29)
Time in inactive intensity (any min)	100–499 Activity counts/min	(12)
Steps in inactive intensity (any min)	Steps detected 100–499 activity counts/min	(12)
Time in low intensity (any min)	100–499 Activity counts/min	(7,13,17)
Time in low intensity (any min)	100–759 Activity counts/min	(43)
Time in lifestyle intensity (any min)	760–2,019 Activity counts/min	(18,33,43,50)
Proportion of time in lifestyle intensity	Proportion of valid wear time at 760–2,019 activity counts/min	(18,33)
Steps in lifestyle intensity (any min)	Steps detected 760–2,019 activity counts/min	(18,50)
Proportion of steps/d in lifestyle activity	Proportion of steps/d at 760–2,019 activity counts/min	(18)
Time in light intensity (any min)	100–573 Activity counts/min	(52)
Time in light intensity (any min)	100–759 Activity counts/min	(33)
Time in light intensity (any min)	100–1,951 Activity counts/min	(10,11,15,23,49)
Time in light intensity (any min)	260–1,951 Activity counts/min	(37,40)
Time in light intensity (any min)	100–2,019 activity counts/min	(52)
Time in light intensity (any min)	500–2,019 Activity counts/min	(12,13,17)
Proportion of time in light intensity	Proportion of valid wear time at 100–759 activity counts/min	(33)
Steps in light intensity (any min)	Steps detected in 500–2,019 activity counts/min	(12)
Time in moderate-intensity activity (any min)	574–4,944 Activity counts/min	(52)
Time in moderate-intensity activity (any min)	2,020–5,998 Activity counts/min	(2,6,16,17,27,30,35,38,52)
Steps in moderate-intensity activity (any min)	Steps detected in 2,020–5,998 activity counts/min	(12)
Time in moderate intensity (modified 10-min bouts)	≥500 Activity counts/min	(21)
Time in moderate intensity (modified 10-min bouts)	760–5,999 Activity counts/min	(44)
Time in moderate intensity (modified 10-min bouts)	≥1,000 Activity counts/min	(21)
Time in moderate intensity (modified 10-min bouts)	≥1,500 Activity counts/min	(21)
Time in moderate intensity (modified 10-min bouts)	≥2,000 Activity counts/min	(21)
Time in moderate intensity (≥10-min bouts)	≥2020 Activity counts/min	(53)
Time in moderate intensity (modified 10-min bouts)	2,020–5,998 Activity counts/min	(2,13,28)
Time in vigorous-intensity activity (any min)	≥4,945 Activity counts/min	(52)
Time in vigorous-intensity activity (any min)	≥5,999 Activity counts/min	(2,7,12,13,16,17,27,30,32,35,38,52)
Time in vigorous (any min)	Not reported	(6)
Steps in vigorous-intensity activity (any min)	Steps detected ≥5,999 activity counts/min	(12)
Time in vigorous intensity (modified 10-min bouts)	≥5,999 Activity counts/min	(2,28,32,44)
Time in MVPA (any min)	≥500 Activity counts/min	(21)
Time in MVPA (any min)	≥574 Activity counts/min	(52)
Time in MVPA (any min)	≥760 Activity counts/min	(37)
Time in MVPA (any min)	≥1,000 Activity counts/min	(21)
Time in MVPA (any min)	≥1,500 Activity counts/min	(21)
Time in MVPA (any min)	≥1,952 Activity counts/min	(10,11,19,22,23,37,40)
Time in MVPA (any min)	≥2,000 Activity counts/min	(21)
Time in MVPA (any min)	≥2,020 Activity counts/min	(2,7,18,33,41,43,50-52)
Proportion of time in MVPA	Proportion of valid wear time ≥2,020 activity counts/min	(18,33)
Time in MVPA (any min outside a ≥10-min bout)	≥760 Activity counts/min	(36)
Time in MVPA (modified 10-min bouts)	≥2,020 Activity counts/min	(1,6,18,28,38,46)
Time in MVPA (in bouts ≥10 min)	≥760 Activity counts/min	(36)
Time in MVPA (in bouts ≥10 min)	≥2,020 Activity counts/min	(35)
Time active	>100 Activity counts/min	(29)
Time in total PA	≥260 Activity counts/min	(40)
Day/wk ≥MVPA	≥2,020 Activity counts/min	(6)
Adherence to PA recommendations (modified 10-min bout)	30 Min of moderate or greater intensity activity on 5 of 7 d	(2,13)
Uncensored (raw) steps/d	Reporting steps as detected	(9,12,13,20,28,51,53)
Censored steps/d	Disqualify steps taken at less than 500 activity counts/min	(9,12,13)
Uncensored (raw) steps/min	Total raw steps accumulated during 1,440 min (24 h or 1 d), divided by time worn	(13)
Censored steps/min	Total steps accumulated during 1,440 min after censoring out steps at an intensity <500 activity counts/min, divided by time worn	(13)
Transitions/d	Total occurrences of when activity counts rose from <100 activity/counts in 1 min to ≥100 activity counts in the subsequent min	(13,15,29)
Basal physical activity	<2,500 Steps/d	(5,12,13)
Limited physical activity	2,500–4,999 Steps/d	(5,12,13)
Sedentary	<5,000 Steps/d	(9,28)
Low active	5,000–7,499 Steps/d	(5,12,13,28)
Low- to somewhat active	5,000–9,999 Steps/d	(9)
Somewhat active	7,500–9,999 Steps/d	(5,12,13,28)
Active to highly active	≥10,000 Steps/d	(9)
Active	10,000–12,499 Steps/d	(5,12,13)
Active	≥10,000 Steps/d	(28)
Highly active	≥12,500 Steps/d	(5,12,13)
Time in incremental cadence bands	0 Steps/min (nonmovement)	(20)
	1–19 Steps/min (incidental movement)	
	20–39 Steps/min (sporadic movement)	
	40–59 Steps/min (purposeful steps)	
	60–79 Steps/min (slow walking)	
	80–99 Steps/min (medium walking)	
	100–119 Steps/min (brisk walking)	
	≥120 Steps/min (all faster human locomotor movements)	
Peak 1-min cadence	Steps/min recorded for the single highest min in a day	(5)
Peak 30-min cadence	Average steps/min recorded for the 30 highest, but not necessarily consecutive, min in a day	(5)

Abbreviation: MVPA, moderate- to vigorous-intensity physical activity.

^a^ Hagströmer et al (43) also reported the number of bouts and accumulated time in each bout in each intensity category (sedentary, low, lifestyle, and moderate or greater). A sedentary bout was defined as more than 5 consecutive minutes within the designated count range, including an allowance for 1 minute above threshold (29). An MVPA bout was defined as 10 or more consecutive minutes within the designated count range, including an allowance for interruption of 1 or 2 minutes below threshold. Metzger et al (38) considered 70% of minutes above threshold in a 10-minute bout; all others considered 80%.

The 2 primary cut point definitions of time spent in MVPA for studies on children or adolescents or both were age-specific values, building on previous research (55), and 3,000 or more activity counts per minute (Table 4). Bouts were defined as any minute, 1 to 4 minutes, 5 to 9 minutes, and 10 or more minutes above threshold, again at times allowing for minimal interruptions below the cut point. Uncensored and censored steps per day were reported. Data were also presented according to a child-specific step-defined graduated index.

**Table 4:** Derived Variables and Cut Point Definitions Used in Studies Published Since 2007 (or Forthcoming) on Accelerometer Data for Children and/or Adolescents, National Health and Nutrition Examination Surveys, 2003–2004 and 2005–2006

Accelerometer-derived variable	Cut point definition	Reference
Mean activity counts/min	Sum of daily activity counts/number of min worn	(2,26,35)
Time in sedentary behaviors	<100 Activity counts/min	(3,30,47)
Sedentary behavior bout	≥30 Min with ≥80% of min <100 activity counts/min (no more than 5 consecutive min ≥100 activity counts/min	(47)
Break min	Within each sedentary behavior bout (defined above), those mins ≥100 activity counts/min	(47)
Time in incidental movement (any movement)	<2,000 Activity counts/min	(48)
Time in low intensity (any movement)	2,000–2,999 Activity counts/min	(48)
Time in low intensity	Between 100 activity counts/min and age-specific cut point definitions (55)	(47)
Time in moderate-intensity activity (any min)	Age-specific cut point definitions (55)	(2,30,38)
Time in moderate-intensity activity (any min)	3000–5,199 Activity counts/min	(48)
Time in moderate- intensity activity (modified 10-min bouts)	Age-specific cut point definitions (55)	(2)
Time in vigorous-intensity activity (any min)	Age-specific cut point definitions (55)	(2,30)
Time in vigorous-intensity activity (any min)	≥5200 Activity counts/min	(48)
Time in vigorous-intensity activity (modified 10-min bouts)	Age-specific cut point definitions (55)	(2)
Time in MVPA (any min)	Age-specific cut point definitions (55)	(2,25,26,30,31,34,35,47,54)
Time in sporadic MVPA (any min <5 min)	Age-specific cut point definitions (55)	(34)
Time in sporadic MVPA (any min <10 min)	Age-specific cut point definitions (55)	(34)
Time in MVPA (any min)	≥1,500 Activity counts/min	(24)
Time in MVPA (any min)	≥3,000 Activity counts/min	(8,39,42)
Time in MVPA (1- to 4-min bouts)	≥3000 Activity counts/min	(39,42,45)
Time in MVPA (modified 5- to 9-min bouts)	≥3000 Activity counts/min	(39,42,45)
Time in MVPA (modified 10-min bouts)	≥3,000 Activity counts/min	(39,42)
Time in MVPA (≥5-min bouts)	Age-specific cut point definitions (55)	(34)
Time in MVPA (≥10-min bouts)	Age-specific cut point definitions (55)	(34)
Time in moderate-intensity activity (modified 10-min bouts)	Age-specific cut point definitions (55)	(2)
Time in total PA	≥2,000 activity counts/min	(8)
Adherence to PA recommendations (children, any min; adolescents, any min and modified 10-min bout)	30 min of moderate- or greater-intensity activity on 5 of 7 d	(2)
Uncensored (raw) steps/d	Reporting steps as detected	(4)
Censored steps/d	Disqualify steps taken <500 activity counts/min	(4)
Sedentary	<10,000 Steps/d (boys aged 6–11); <7,000 steps/d (girls aged 6–11)	(4)
Low active	10,000–12,499 Steps/d (boys aged 6–11); 7,000–9,499 steps/d (girls aged 6–11)	(4)
Somewhat active	12,500–14,999 Steps/d (boys aged 6–11); 9,500–11,999 steps/d (girls aged 6–11)	(4)
Active	15,000–17,499 Steps/d (boys aged 6–11); 12,000–14,499 steps/d (girls aged 6–11)	(4)
Highly active	≥17,500 Steps/d (boys aged 6–11); ≥14,500 steps/d (girls aged 6–11)	(4)

Abbreviations: PA, physical activity; MVPA, moderate- to vigorous-intensity PA.

## Discussion

An obvious advantage of NHANES accelerometer data is that they reflect objectively measured behaviors that can be examined, compared, and related to other NHANES data. NCI-supplied SAS syntax has facilitated analysis of these data. When studies in our review did not explicitly report some decision rules, they frequently reported use of SAS syntax or they cited previous methods that had used this tool, suggesting that SAS syntax was likely applied. Clearly, researchers have treated, analyzed, and reported PAM data in nonstandardized ways, which compromises the ability to make comparisons among studies. This lack of uniformity is perhaps most apparent in the multiple cut point definitions of time spent in MVPA. Inconsistent approaches will impede the ability to track behaviors over time in the United States and compare US behaviors with behaviors in other countries.

The intent of this review was neither to judge researchers’ decisions about examining PAM data nor to make pronouncements on the most appropriate strategies. Unique research questions may require equally unique analytical approaches; some degree of inconsistency must be tolerated if scientific discovery is to be encouraged. That being said, consumers (including the research community) of these data must be informed about inconsistencies, especially when different cut point definitions are used for similarly named variables.

One of the primary challenges to implementing a measure of accelerometer-based physical activity in a study is ensuring compliance with monitoring protocols. The 2003–2004 and 2005–2006 NHANES protocols asked participants to remove the accelerometer only during sleep and water-based activities (eg, swimming, showering, bathing). Conclusions about accelerometer-based behavior are affected by definitions of nonwear time and a valid day (56). Most studies included in our review defined nonwear time as 60 minutes or more of consecutive zeros. Differences between allowances for interruptions may simply represent reporting discrepancies, especially because many of the studies catalogued in Table 2 also reported using SAS syntax (or cited methods of previous work that did). The NCI decision to use 60 minutes of consecutive zeros to identify nonwear time was based on research by Mâsse et al (57). These researchers demonstrated that sample sizes were optimized when nonwear time was defined as 60 minutes, rather than 20 minutes, of continuous zeros. A recent study indicated that 90 minutes of continuous zeros may provide more accurate estimates of time in sedentary and active behaviors (58).

Researchers were also almost perfectly consistent in defining a valid day as 10 or more hours of wear time, which is also the definition provided by SAS syntax. (In 2 of the 4 studies that did not report nonwear criteria, we assumed that they used this definition because they reported using SAS syntax.) Mâsse et al (57) compared results of studies that used different definitions of a valid day, and although they did not recommend a specific number of hours of wear time to define a valid day, they noted that the strictest requirement (≥12 h/d) negatively affected sample size. They also speculated that stricter requirements might unduly limit inclusion of inactive people, thereby affecting overall data distribution. The popularity of using 10 or more hours of wear time to define a valid day is likely due to numerous factors: 1) it was a component of one of the decision-rule algorithms evaluated by Mâsse et al (57); 2) it was used in the seminal NHANES PAM data publication (2); and 3) it was built into the NCI-provided SAS syntax to accompany the NHANES accelerometer data. Using 2005–2006 NHANES data, Tudor-Locke et al (7) showed that, as population estimates of nonwear increase, all other time in intensity (eg, MVPA) and volume (ie, activity counts/d, steps/d) indicators decrease to some degree, but the negative effect is most pronounced on estimates of time spent in sedentary behavior. Nonwear time is more likely to reflect time spent in sedentary behaviors than in active behaviors. Mâsse et al (57) reached a similar conclusion: varying minimal wear-time requirements primarily affected minutes of inactivity (their preferred term). Others concluded the same (22). The effect of reduced wear time on estimates of sedentary behavior should not differ by age. Although there is apparent consensus that 10 or more hours of wear time is adequate to define a valid day, a 24-hour wear-time protocol would remove much ambiguity from analysis (59).

Regardless of how scientists have analyzed NHANES accelerometer data, however, it remains clear that the US citizenry is not very active. Troiano et al (2) reported that less than 5% of adults achieve public health guidelines, although this low estimate may be an artifact of the minimal bout criterion and a cut point definition that was based primarily on locomotor activities. Matthews et al (3) reported that more than 50% of monitored time is spent in sedentary behaviors. Tudor-Locke et al (12) reported that NHANES adults took an average of approximately 6,500 steps/day (considered “low active” on a pedometer-based scale). Using identical accelerometer models and analytic methods to directly compare Swedish data with NHANES data, Hagströmer et al (43) showed that the amount of time spent in MVPA was not uniformly greater in Sweden than in the United States, even though Sweden has a population that is generally considered to be quite active.

Although we limited our online search to English-language articles indexed in PubMed, we are confident that this search engine was the best one for identifying articles on NHANES accelerometer data. This free resource is maintained by the National Center for Biotechnology Information at the US National Library of Medicine, which is located at the National Institutes of Health. We included only 1 article that was not indexed in PubMed. Our search spanned 2007 (the year that these data were released) through 2011; however, we acknowledge there may be additional forthcoming articles that we did not identify. This review necessarily represents a limited time frame.

NHANES accelerometer data represent an important public use resource for researchers and practitioners engaged in designing and directing health programs and services and developing public health policy. This review was undertaken to summarize existing research that has used these data. The studies we identified bear evidence of the multiple and diverse uses of these data, and we can anticipate that they will continue to be used in epidemiologic and health sciences research. We hope that the resulting catalog of accelerometer decision rules, derived variables, and cut point definitions used to analyze these NHANES data serves as a useful starting point for future researchers to consider as they plan and report their own analyses.
